# Dose‐dependant acute or subacute disease caused by *Burkholderia pseudomallei* strain NCTC 13392 in a BALB/c aerosol model of infection

**DOI:** 10.1111/jam.14396

**Published:** 2019-08-09

**Authors:** S.G.P. Funnell, J.A. Tree, G.J. Hatch, S.R. Bate, G. Hall, G. Pearson, E.L. Rayner, A.D.G. Roberts, J. Vipond

**Affiliations:** ^1^ National Infection Service Public Health England (PHE) Salisbury Wiltshire UK

**Keywords:** aerosol, BALB/c, Burkholderia, dose, Melioidosis, pseudomallei, subacute

## Abstract

**Aims:**

The goal of this study was to examine, for the first time, the virulence and pathogenicity of aerosolized *Burkholderia pseudomallei*, strain NCTC 13392, in BALB/c mice in order to develop an animal model for testing novel medical countermeasures (MCMs) for the treatment of human acute and subacute (a disease state between acute and chronic) melioidosis.

**Methods and Results:**

BALB/c mice were exposed to varying doses of aerosolized bacteria. Acute disease was seen in animals exposed to a very‐high dose (≥10^3^ CFU per animal) and death occurred 3–4 days postchallenge (pc). Bacteria were detected in the lungs, liver, kidney and spleen. In contrast, animals exposed to a low dose (<10 CFU per animal) survived to the end of the study (day 30 pc) but developed weight loss, a bacterial tissue burden and increasing clinical signs of infection from day 20 pc onwards, mimicking a subacute form of the disease. Pathological changes in the tissues mirrored these findings.

**Conclusions:**

This proof of concept study has shown that *B. pseudomallei* strain NCTC 13392 is virulent and pathogenic in BALB/c mice, when delivered by aerosol. By varying the doses of aerosolized bacteria it was possible to mimic characteristics of both human acute and subacute melioidosis, at the same time, within the same study.

**Significance and Impact of the Study:**

*Burkholderia pseudomallei*, the aetiological agent of melioidosis, causes a serious and often fatal disease in humans and animals. Novel MCMs are urgently needed for both public health and biodefense purposes. The present model provides a useful tool for the assessment and evaluation of new MCMs (e.g. therapeutics and vaccines) and offers the potential for testing new treatments for both subacute to chronic and acute melioidosis prior to human clinical trials.

## Introduction

*Burkholderia pseudomallei*, the aetiological agent of melioidosis, causes a serious and often fatal disease in humans and animals (Titball *et al.* 2008). Despite the global widespread geographical occurrence of naturally acquired melioidosis, particularly in Southeast Asia and Northern Australia (Limmathurotsakul *et al.* 2016), there is no licensed vaccine and antibiotic therapy is problematic because *B. pseudomallei* is intrinsically resistant to many antibiotics (Wiersinga *et al.* 2006). Concerns over the use of *B. pseudomallei* as a biological weapon has resulted in this pathogen being classified as a Tier 1 Select Agent by the United States Federal Select Agent program (http://www.selectagents.gov/). Novel therapeutics and vaccines, otherwise known as medical countermeasures (MCMs), are needed for both public health and biodefense purposes.

Melioidosis can affect individuals with pre‐existing health conditions such as diabetes mellitus (50% of cases) (Wiersinga *et al.* 2006). The disease can present a broad range of symptoms, with both acute and chronic phases. Acute disease can be severe with symptoms including fever, weight loss, pneumonia, sepsis and death (Currie *et al.* 2000; Boruah *et al.* 2013; Cheng *et al.* 2013; Jin and Ning, 2014). Chronic infection can be less severe and may present as abscesses distributed in single or multiple sites, including the spleen, lung, liver, joints and central nervous system (Wong *et al.* 1995; Kunnathuparambil *et al.* 2013; Liang *et al.* 2016). In addition, a latent or reactivated form of disease may appear years later: the longest recorded incubation period being 62 years (Ngauy *et al.* 2005). Humans can be infected naturally with *B. pseudomallei* following bacterial inoculation, inhalation or ingestion (Peacock *et al.* 2012). The route of infection and severity of disease are linked, with inhalation associated with a more rapid disease course (Titball *et al.* 2008).

Treatment for melioidosis involves an intensive intravenous antibiotic infusion phase for a minimum of 10–14 days followed by a lengthy eradication phase of 3–6 months of oral antibiotic therapy (Pitman *et al.* 2015). Given the multidrug‐resistant nature of *B. pseudomallei* to common antibiotics and the lengthy treatment regime for melioidosis, new MCMs are urgently needed. However, the protective efficacy of new therapeutics and vaccines are difficult to assess in Phase III human clinical trials because of the low incidence of naturally acquired *B. pseudomallei* in most parts of the world. It is also difficult to establish the exact route of entry of infection in human trials conducted in endemic countries. For this reason, well‐characterized animal models of infection, with well‐defined endpoints relevant to human disease will help evaluate the efficacy of novel MCMs for use in humans.

The mouse model of melioidosis is commonly used for prescreening the effectiveness of novel therapeutics before they are tested in larger animal models. Prior to evaluation, however, the decision about which mouse strain, the route of infection and which strain of *B. pseudomallei* to use is challenging as a variety of studies have been reported (Jeddeloh *et al.* 2003; Titball *et al.* 2008; Lever *et al.* 2009; West *et al.* 2010; Conejero *et al.* 2011; Thomas *et al.* 2012; West *et al.* 2012; Lafontaine *et al.* 2013; Massey *et al.* 2014; Welkos *et al.* 2015; Trevino *et al.* 2018),. Some studies show BALB/c mice to be more sensitive to infection with *B. pseudomallei* and thus help reflect acute human infection, while C57BL6 have been shown to be more resistant and thus mimic chronic infection (Leakey *et al.* 1998; Hoppe *et al.* 1999; Liu *et al.* 2002; Tan *et al.* 2008; Bearss *et al.* 2017; Trevino *et al.* 2018). Conversely, a chronic model of infection in BALB/c mice has been recently reported which utilizes a strain of *B. pseudomallei* 1106a, which has low virulence, delivered intraperitoneally (Amemiya *et al.* 2015). Among the reported mouse studies, a variety of delivery routes have been used (aerosol, intratracheal, intranasal, intravenous and intraperitoneal); the aerosol challenge is the most useful route, if evaluating MCMs for biodefense purposes. Choosing a representative *B. pseudomallei* challenge strain is problematic because there is a high degree of genetic and phenotypic variability among *B. pseudomallei* isolates (Ronning *et al.* 2010; Van Zandt *et al.* 2012; Sarovich *et al.* 2014; Welkos *et al.* 2015; Trevino *et al.* 2018). There is also a lack of understanding of the impact of strain variation on virulence, pathogenicity and mortality in mice; although some studies seek to further define this (Massey *et al.* 2014; Sahl *et al.* 2015). New information about novel strains adds to this body of literature, thus further characterizing the murine experimental model of melioidosis and its alignment to human disease.

The purpose of the present proof of concept study was to characterize, for the first time, the virulence and pathogenicity of *B. pseudomallei* strain NCTC 13392 (Sahl *et al.* 2013) in a BALB/c aerosol infection model. This novel murine model provides the opportunity to study subacute disease (a disease state between acute and chronic) alongside acute disease in the same experiment following exposure to different aerosolized doses of bacteria. Initially a 14‐day virulence study was performed followed by a 30‐day pathogensis study; clinical score, weight, bacterial burden and histopathology are reported.

## Materials and methods

### Bacterial strain

*Burkholderia pseudomallei* strain NCTC 13392, was obtained from PHE, Culture Collections, Porton Down, Salisbury, UK. This strain was originally isolated from a septicaemic, diabetic patient with fatal melioidosis, in Khon Kaen, Thailand in 1996 at the same time as strain K96243 (Sahl *et al.* 2013). Whole genome sequencing was performed, on the isolate used in this study, using an Illumina HiSeq system.

### Experimental animals

Healthy BALB/c female mice (purchased from UK Home Office accredited suppliers, Charles Rivers UK, Ltd and Envigo CRS Ltd, UK) were used, aged 10–12 weeks and weighing 18 g or more at the time of challenge. They were group housed in cages in accordance with the UK Home Office *Code of Practice for the Housing and Care of Animals Bred, Supplied or Used for Scientific Procedures* (December 2014) at Advisory Committee on Dangerous Pathogens (ACDP) containment level 3 and provided with access to food and water *ad libitum*. All experimental work was conducted under the authority of a UK Home Office approved project licence that had been subject to local ethical review at PHE Porton Down by the Animal Welfare and Ethical Review Body (AWERB) as required by the *Home Office Animals (Scientific Procedures) Act 1986*. Before the start of each experiment, animals were randomly assigned to the treatment groups in order to minimize bias.

### Culture conditions

Each culture of *B. pseudomallei* NCTC 13392 was prepared from a fresh vial of working stock on plates of Luria–Bertani Agar (LBA). Plates were incubated for 24–48 h at 37°C. Growth from one LBA plate was suspended in 10 ml of Luria–Bertani Broth (LBB) to yield an undiluted primary seed. The primary seed was further diluted in LBB to achieve a 100 ml culture with an optical density (OD) OD_600_ of 0·1. This was incubated with orbital shaking at 175 rev min^−1^ for 24 h at 37°C and used in the aerosol inoculation procedures.

### Aerosol procedures

The 24 h LBB broth culture of *B. pseudomallei* NCTC 13392 was diluted in phosphate‐buffered saline (PBS) to achieve the target presented doses which were nominally grouped as follows: very‐high dose (≥10^3^ CFU per animal), high dose (10^2^ to <10^3^ CFU per animal), medium dose (10 to <10^2^ CFU per animal) and low dose (<10 CFU per animal).

All mice were exposed to a small particle aerosol (mass medium aerodynamic diameter ~1·5 *µ*m) of bacteria in an AeroMP‐Henderson apparatus. The challenge aerosol was generated using a three‐jet Collison Nebuliser (BGI, Inc., Waltham, MA); the aerosol was mixed with conditioned air in the spray tube and delivered to the nose of each animal through an exposure tube in which nonanesthetized mice were held in restraint tubes. Samples of the aerosol were collected in PBS using an AGI30 glass impinger (Ace Glass, Inc., Vineland, NJ), and the mean particle size was determined with an aerodynamic particle sizer (TSI Instruments, Ltd., High Wycombe, UK). These processes were controlled and monitored from an AeroMP management platform (Biaera Technologies, Hagerstown, MD). The control animals were challenged with an aerosol produced from LBB/PBS in the same ratio as the bacterial challenge preparation. The presented dose was calculated using the results of bacterial enumeration of aerosol samples taken throughout exposure and estimates of respired volume calculated using Guyton’s formula (Guyton, 1947).

### Virulence study

In order to determine the virulence of *B. pseudomallei* NCTC 13392, an initial short‐term (14 day) survival study using 35 BALB/c mice was performed. Very high, high and medium doses were delivered by aerosol to three separate groups of 10 mice. Control mice (*n* = 5) were sprayed with sterile LBB/PBS broth. At 14 days postchallenge (pc), the surviving animals were killed humanely with an intraperitoneal overdose of pentobarbitone (Dolelethal, Vétoquinol UK Ltd, 140 mg kg^−1^).

### Pathogenesis study

In order to determine the pathogenicity of *B. pseudomallei* NCTC 13392, a longer term (30 day) study was conducted using 60 BALB/c mice. Three aerosol doses were delivered to three groups of mice: very high (*n* = 15 animals), high (*n* = 20) and low (*n* = 20). Groups of five mice were euthanized on scheduled days. For the very‐high‐dose group mice, were scheduled for euthanasia on day 1 (*n* = 5), day 3 (*n* = 5) and day 4 (*n* = 5). For the high‐dose group, mice were euthanized on day 3 (*n* = 5), day 10 (*n* = 5), day 20 (*n* = 5) and day 30 (*n* = 5); a similar schedule was used for the low‐dose group. The control animals (*n* = 5) were sprayed with sterile LBB/PBS and euthanized on day 14. On the day of euthanasia, organs were collected for bacterial burden and histopathological examination.

### Clinical and euthanasia observations

Animals were weighed at the same time of day from the day before infection until the end of the experiment, or until death or euthanasia. Animals were examined twice daily for clinical signs of disease and scores were recorded using a ‘clinical observation score’. Monitoring of animals increased to at least four times daily during critical periods in the study (at times when septicaemia was likely to occur or as soon as clinical signs of septicaemia were detected). Clinical signs of disease were assigned a score based upon the following criteria: healthy, 0; ruffled fur, 2; arched back, 3; eyes shut, 3; immobile, 9; euthanasia or death in cage, 10. Clinical scores were not used to trigger euthanasia. In order to meet the requirement of the project license, three criteria for immediate euthanasia were used: (i) immobility defined as a lack of movement even after stimulus such as handling, (ii) neurological signs including repetitive or unusual movement, (iii) weight loss of more than 20% from baseline for more than 24 h. If any animal reached any of these three euthanasia criteria, they were immediately euthanized using a UK Home Office approved Schedule 1 procedure.

### Bacteriology

At necropsy, the same tissues were collected from each animal: the left lobe of the lung, the apical section of the spleen, a section from the right lateral lobe of the liver and the left section of the kidney. These tissues were frozen below −50°C. Tissue was thawed and homogenized in LBB with 1·4‐mm ceramic beads in a Precellys24 tissue homogenizer (Bertin Technologies, Villeurbanne, France). Serial 10‐fold dilutions of tissue homogenates were plated on LBA. Bacterial colonies were counted after incubation for 48 h at 37°C.

### Pathology studies

Throughout the pathogenesis study, gross abnormalities were recorded. At necropsy, following the collection of samples for bacteriology, samples of lung, liver and kidney were collected for histopathological examination. They were placed in 10 % neutral buffered formalin, processed routinely to paraffin wax and 3–5‐µm sections cut and stained with haematoxylin and eosin (H and E).

### Statistical analysis

The Kaplan–Meier method was used to plot the survival data from the virulence study. Differences in the number of CFUs in tissues, on different days, were determined by performing Student’s *t*‐tests using 95% confidence interval on log_10_ transformed data. All combinations were tested, that is, day 10 *vs* day 20, day 20 *vs* day 30, etc.) Differences that were significant at *P* < 0·05 are reported. All analyses were done using Minitab version 16.

## Results

### Whole‐genome sequencing

Whole‐genome sequencing of the isolate used in this study was conducted by PHE for verification purposes (data not shown). The sequence showed identity (within 6–8 SNPs) with the strain supplied by PHE to Northern Arizona University, Center for Microbial Genetics and Genomis, Flagstaff, Arizona, USA who first published the genome of NCTC 13392 in 2013 (Sahl *et al.* 2013).

### Virulence study

**Figure 1:**
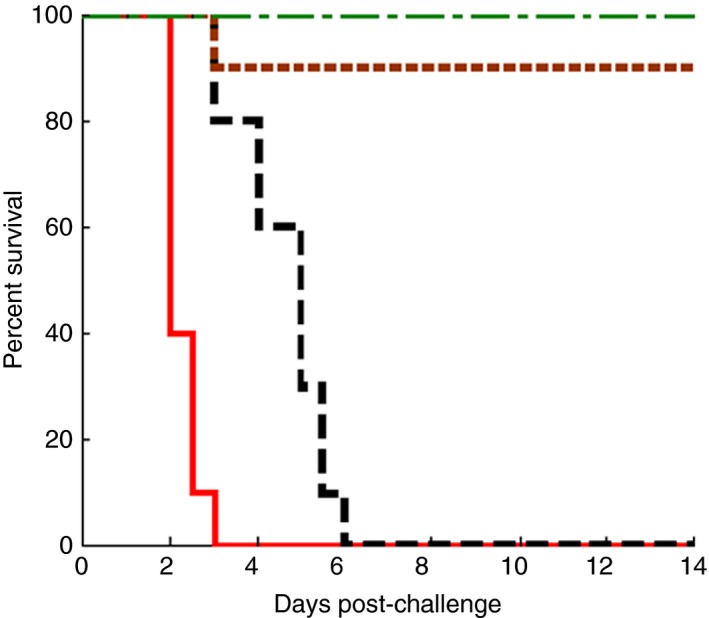
Virulence study: Fourteen‐day survival rate of BALB/c mice infected with aerosolized *Burkholderia pseudomallei*. BALB/c mice were infected with different doses; very high (3·33 × 10^3^ CFU per animal) (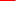) (solid line), high (7·85 × 10^2^ CFU per animal) (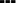) (dashed line) and medium (6·85 × 10^1^ CFU per animal) (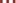) (dotted line) doses of *B. pseudomallei* and the 14‐day survival rates of mice were plotted, using the Kaplan–Meier Method. The control group (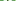) (dash‐dot‐dash line). [Colour figure can be viewed at wileyonlinelibrary.com]

Animals exposed to the very‐high dose (3·33 × 10^3^ CFU per animal) of aerosolized *B. pseudomallei* NCTC 13392 died (10/10) within 3 days (Fig. 1). Those challenged with the high dose (7·85 × 10^2^ CFU per animal) survived longer, but died by day 6 pc. In the group given the medium dose (6·85 × 10^1^ CFU per animal), 9 of 10 animals survived beyond the end of the experiment at 14 days. Information gained from this virulence study was used to determine the doses for the pathogenesis study.

### Pathogenesis study

Animals exposed to the very high dose (3·04 × 10^3^ CFU per animal) of aerosolized *B. pseudomallei* NCTC 13392 where scheduled to be euthanized on days 1 (*n* = 5), 3 (*n* = 5) and 4 (*n* = 5) pc; however, some animals in the day 4 group met euthanasia criteria early, on day 3, and were thus included in the day 3 group (*n* = 8). This meant only two animals remained and they were euthanised on day 4, as planned. Those challenged with the high dose (1·11 × 10^2^ CFU per animal) survived to the end of the study (day 30). Those animals in the low‐dose group (7·69 × 10^0^ CFU per animal) also survived to the end of the study.

### Clinical signs

**Figure 2:**
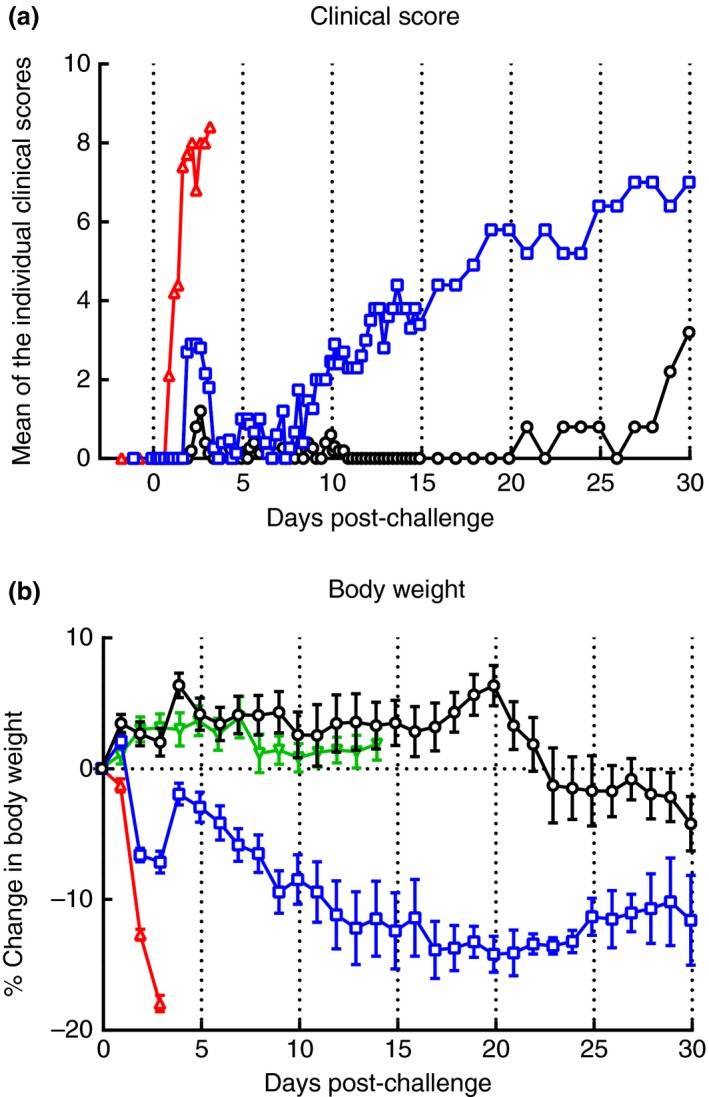
Pathogenesis study: Individual clinical scores and change in percentage body weight after aerosol challenge with *Burkholderia pseudomallei* or vehicle control. Bacteria were presented in an aerosol (via a Henderson apparatus using three different doses) to BALB/c mice (*n* = 60). The presented doses were: very high (3·04 × 10^3^ CFU per animal) (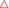); high (1·11 × 10^2^ CFU per animal) (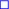) and low (7·69 × 10^0^ CFU per animal) (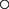). Mice (*n* = 20 per dose at the start of the experiment) were monitored for clinical signs of infection and body weight changes. Results are expressed as (a) mean of the individual clinical scores (NB, control group (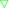) had a zero clinical score throughout the study, data not plotted), and (B) the mean percentage (%) change in body weight compared to baseline levels, ±1 SE. [Colour figure can be viewed at wileyonlinelibrary.com]

In the pathogenesis study, all 15 animals challenged with a very high dose of *B. pseudomallei* developed high clinical scores (8·4) and progressed rapidly to death (Fig. 2a). Ruffled fur was present from 24 h pc, followed by lethargy with arched backs which progressed to immobility or death by 3–4 days in all the animals. Animals (*n* = 20) exposed to the high dose developed clinical signs at day 3 pc that abated slightly until day 5 when signs re‐emerged and the health of animals deteriorated slowly, with ruffled fur and arched backs being common (Fig. 2a). The mean individual clinical score increased to 7 by the end of the study (day 30 pc), when the remaining animals (5/5) were killed humanely. Animals (*n* = 20) exposed to the low dose displayed few clinical signs initially (2/5 animals had ruffled fur); these abated until day 20 pc. From day 20 pc, the mean individual clinical score began to increase, reaching 3·2 on day 30 pc (5/5 animals had ruffled fur and 2/5 animals had arched backs). Animals in the control group (5/5), killed humanely on day 14 pc, remained free of clinical signs throughout the study (data not shown).

### Body weight

Animals in the very‐high‐dose group lost upto 18% of body weight, compared to baseline levels, within 3 days following challenge (Figure 2b). Animals challenged with the high dose lost 7% of their body weight by day 3 but then regained (5%) weight quickly within 24 h, afterwards there was a slow, consistent weight loss for the next 15 days. Weight change in the low‐dose group was similar to animals in the control group (Figure 2b) up to day 14. On day 20, an increase in weight (6%) was observed compared to baseline levels; however, after this animals lost 10% of their body weight by day 30 (4% lower than at the start of the experiment).

### Bacteriological studies

**Figure 3:**
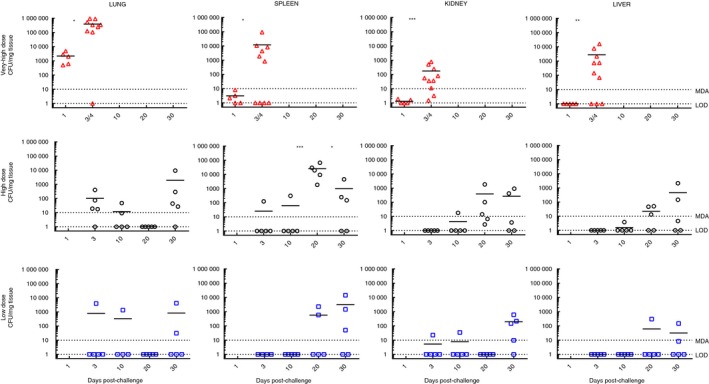
Pathogenesis study: Bacterial loads in the lungs, kidney, spleen and liver. Bacteria were presented in an aerosol (via a Henderson apparatus using three different doses) to BALB/c mice (*n* = 60). The presented doses were: very high (3·04 × 10^3^ CFU per animal) (∆); high (1·11 × 10^2^ CFU per animal) (□) and low (7·69 × 10^0^ CFU per animal) (○). At predetermined time points postinoculation (see Materials and methods), mice (*n* = 5) were euthanized and various postmortem tissues collected. The tissues were stored frozen and subsequently thawed and homogenized so that bacterial tissue burdens could be determined. Individual results are plotted plus geometric mean, bar. For the very‐high‐dose group, the data for days 3 and 4 have been plotted together for graphical purposes to enable a comparison across all data sets (the study terminated on days 3/4). For the low‐ and high‐dose groups, animals were not culled on day 1. For graphical presentation all samples that were below the limit of detection (LOD) were assigned a nominal value equivalent to the LOD (1·0 CFU per mg tissue). The minimum detectable amount varied between tissues but is shown for illustration purposes only. Significant differences between the tissue loads are indicated by asterisk *P < 0·05, **P < 0·01 and ***P < 0·001, the data sets acquired on different days were compared, for example, for the very‐high‐dose group day 1 *vs* day 3/4 were compared for each tissue type. [Colour figure can be viewed at wileyonlinelibrary.com]

In the very‐high‐dose group, of the four tissues tested, the highest mean concentration of bacteria was in the lung (2·2 × 10^3^ CFU per mg) on day 1 (Figure 3). By days 3–4 pc this significantly (*P* < 0·05) rose to an average of 3·9 × 10^5^ CFU per mg (9/10 animals). In the spleen, kidney and liver (10/10 animals), bacteria were present at low levels (≤8 CFU per mg) on day 1, increasing significantly (*P* < 0·05) by days 3–4 to means of 1·2 × 10^4^, 1·9 × 10^2^ and 3·0 × 10^3^ CFU per mg tissue respectively.

In the high‐dose group, bacteria were detected in 4 of 5 animals in the lung and in 1 of 5 animals in the spleen on day 3 pc. Bacterial numbers in the spleen (5/5) increased significantly (*P* < 0·001) peaking on day 20 (average 2·5 × 10^4^ CFU per mg), whereas bacterial counts decreased (5/5) in the lung by day 20 (≤1 CFU per mg). Between days 20 and 30 pc, there was a rise in bacterial counts in the lung, peaking at an average 1·9 × 10^3^ CFU per mg. In the spleen a significant (*P* < 0·05) reduction in the mean number of bacteria was observed (mean, 9·7 × 10^2^ CFU per mg). Throughout the course of the study there was a gradual rise in the number of bacteria in the kidney and liver; by day 30 pc these had reached mean levels of 2·7 × 10^2^ and 4·6 × 10^2^ CFU per mg respectively.

In the low‐dose group, only 1 of 5 animals had detectable levels of bacteria in the lungs (3·9 × 10^3^ CFU per mg) at 3 days pc and 1 of 5 animals on day 10 pc (1·3 × 10^3^ CFU per mg). Bacteria were not detected on day 20 pc in 5 of 5 animals (≤1 CFU per mg, limit of detection) in the lungs. However, by day 30 pc, infection was re‐established in the lungs of 2 of 5 animals (mean 2 × 10^3^ CFU per mg, *n* = 2). Similarly in the kidney, bacteria (2·2 × 10^1^ CFU per mg) were present in 1 of 5 animals on day 3 pc and in 1 of 5 animals (3·4 × 10^1^ CFU per mg) on day 10 pc. Bacteria were not detected on day 20 pc, but again infection was re‐established in 4 of 5 animals by day 30 (2·4 × 10^2^ CFU per mg, *n* = 4). No bacteria were detected in the liver or spleen on days 3 and 10 pc; however, by day 20, bacteria were found in 2 of 5 animals (mean 1·4 × 10^3^ CFU per mg, n = 2) in the spleen and in 1 of 5 animals in the liver 3 × 10^2^ CFU per mg). By day 30 pc, 3 of 5 and 2 of 5 animals had bacteria in the spleen and liver respectively. *Burkholderia pseudomallei* were not isolated from the tissues of control animals.

### Histopathology findings

The findings for all groups are summarized in Table 1. Five lesion types were seen in lung that were associated with infection with *B. pseudomallei*. These comprised: parenchymal, multifocal pyogranulomatous inflammation, with either central necrosis (A), or the lesions were non‐necrotic (B); patchy, interstitial, histiocytic inflammation, variably accompanied by neutrophils (C); bronchial and bronchiolar inflammatory cell infiltrates (D); and bronchial and bronchiolar epithelial cell necrosis (E). In the spleen, multifocal, necrotizing splenitis with neutrophils ± lymphocytes, with cellular degeneration (necrosis/apoptosis) (F), and large, well‐demarcated pyogranulomatous foci (abscesses) (G), were observed. In the liver, two lesions were noted, namely random, mulitfocal, necrotizing hepatitis ± neutrophils (H), and hepatocyte degeneration and focal inflammatory cell infiltrate (I).

**Table 1:** Histological features of pathogenesis study. A summary of treatment‐related changes in the lung, spleen and liver on different days. Number of animals with treatment‐related changes/total number of animals. No changes were observed in the kidney of any animal in any group

Organ	Lesion	Exposure dose (no. of animals with treatment‐related changes/total no. of animals)											
		Very high dose				High dose				Low dose			
		Days postchallenge											
		1	3	4	–	3	10	20	30	3	10	20	30
Lung	Parenchymal, multifocal, necrotizing, pyogranulomatous inflammation	0/5	3/8	2/2	–	2/5	0/5	1/5	1/5	1/5	1/5	1/5	0/5
	Parenchymal, multifocal, pyogranulomatous inflammation	5/5	5/8	0/2	–	3/5	0/5	1/5	1/5	0/5	1/5	1/5	0/5
	Patchy, interstitial histiocytic inflammation ± neutrophils	2/5	5/8	2/2	–	3/5	2/5	5/5	4/5	2/5	2/5	3/5	4/5
	Bronchial/bronchiolar inflammatory cell exudates	4/5	7/8	2/2	–	3/5	0/5	1/5	0/5	2/5	1/5	1/5	0/5
	Bronchial/bronchiolar epithelial cell necrosis	1/5	3/8	0/2	–	0/5	0/5	1/5	0/5	2/5	1/5	1/5	0/5
Spleen	Multifocal, necrotizing splenitis with neutrophils ± lymphocyte necrosis/apoptosis	0/5	2/8	2/2	–	0/5	0/5	1/5	0/5	4/5	1/5	1/5	4/5
	Large, well‐demarcated pyogranulomatous foci (abscess)	0/5	0/8	0/2	–	0/5	2/5	5/5	3/5	0/5	2/5	4/5	3/5
Liver	Random, multifocal, necrotizing hepatitis ± neutrophils	1/5	4/8	2/2	–	2/5	3/5	2/5	4/5	1/5	3/5	1/5	2/5
	Hepatocyte degeneration and focal inflammatory cell infiltrate	0/5	0/8	0/2	–	3/5	5/5	4/5	4/5	3/5	4/5	4/5	4/5

### Very‐high‐dose group

**Figure 4:**
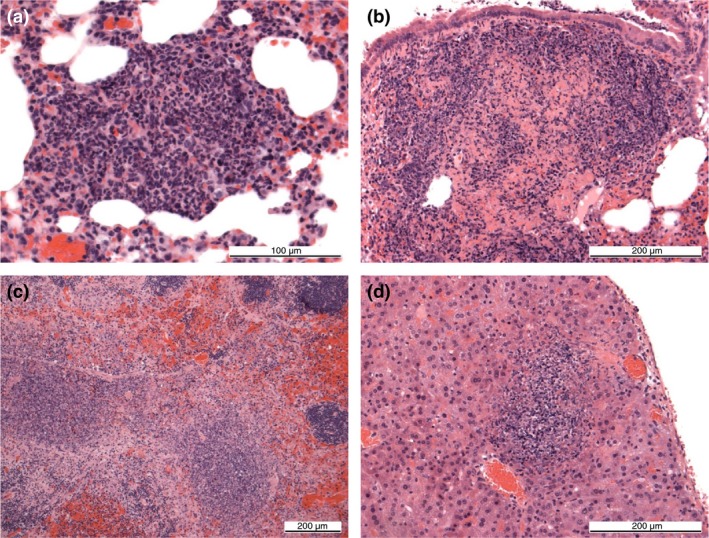
Pathogenesis study: Examples of lesions. (a) Lung, very‐high‐dose group, 1 day pc. Focal, pyogranulomatous inflammation. (b) Lung, very‐high‐dose group, 3–4 days pc. Focal, necrotizing, pyogranulomatous inflammation. (c) Spleen, very‐high‐dose group, 3–4 days pc. Multifocal, coalescing, necrotizing splenitis in the red and white pulp. (d) Liver, very‐high‐dose group, day 1 pc. Focal, necrotizing hepatitis (All sections stained with haemotoxylin and eosin). [Colour figure can be viewed at wileyonlinelibrary.com]

No animal survived beyond day 4 pc. In the lung at day 1, lesions B–E were present with varying frequency (Fig. 4a). Necrosis was not observed within pyogranulomas (A) until day 3 pc (Fig. 4b). In the spleen, changes were absent on day 1 pc. By days 3 and 4 pc, necrotizing splenitis (F) was the only lesion type observed, and present in a proportion of animals (Fig. 4c). In the liver, necrotizing hepatitis (H) was seen in a proportion of animals at each time point pc (Fig. 4d).

### High‐dose group

**Figure 5:**
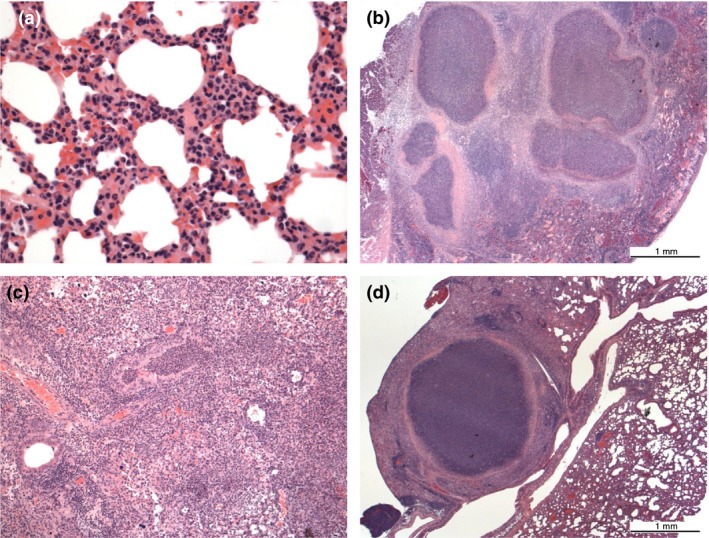
Pathogenesis Study: Examples of lesions. (a) Lung, high‐dose group, day 10 pc. Patchy, interstitial, histiocytic cell infiltration of alveolar walls, with scattered neutrophils. (b) Spleen, high‐dose group, 10 days pc. Coalescing, large, well‐demarcated, pyogranulomatous foci (abscesses). (c) Lung, low‐dose group, 3 days pc. Prominent, bronchial and bronchiolar inflammatory cell exudates, comprising primarily neutrophils, filling lumena (arrow), surrounded by necrotizing, pyogranulomatous inflammation. (d) Lung, low‐dose group, 10 days pc. Well‐demarcated, pyogranuloma (abscess) surrounded by fibrosis and inflammatory cells (All sections stained with haemotoxylin and eosin). [Colour figure can be viewed at wileyonlinelibrary.com]

Lesions were assessed at 3, 10, 20 and 30 days pc. Lung lesions A and B, namely pyogranulomatous inflammation in the presence or absence of necrosis, were common at day 3 pc and less common subsequently (Fig. 5a). In contrast, lesions C–E were observed frequently at the later time points. In the spleen, necrotizing splenitis (F) was limited to one animal at day 20 pc. Pyogranulomas (G) were not seen at day 3 pc, but were common at days 10, 20 and 30 (Fig. 5b). In the liver, both lesion types H and I were seen frequently at all time points pc.

### Low‐dose group

Lesions were assessed at 3, 10, 20 and 30 dpc. In the lung, interstitial histiocytic inflammation (C) was the most frequently observed lesion type at all time points. The remaining changes were seen occasionally (Figure 5c,d). In the spleen, both splenitis (F) and pyogranulomas (G) were present at all time points. In the liver, both lesion types H and I were seen frequently at all time points pc. Histopathological changes were not detected in the kidney in any animal in any group.

## Discussion

Well‐characterized animal models are needed to better understand the pathogenic process of infection by aerosolized *B. pseudomallei* (Massey *et al.* 2014). Animal models are also needed to help evaluate the efficacy of novel therapeutics because Phase III human clinical trials are difficult to perform. This study reports on, for the first time, the virulence and pathogenicity of *B. pseudomallei* NCTC 13392 in BALB/c mice where both acute and subacute disease were observed following challenge with different aerosolized doses of bacteria.

Acute disease was observed when BALB/c mice were challenged with a very high dose (>10^3^ CFU per animal) of *B. pseudomallei* NCTC 13392. Animals succumbed to the disease very quickly and died within 3–4 days following exposure. Acute disease was characterized by mean high individual clinical scores of 8·4 (comprising ruffled fur, arched backs and lethargy) and weight loss (up to 18%). In people, acute disease is characterized by a range of symptoms including weight loss, fever, pneumonia, sepsis and death (Currie *et al.* 2000; Boruah *et al.* 2013; Cheng *et al.* 2013; Jin and Ning, 2014). In people, the lung is the most commonly affected organ. Changes in the lung, described in the current study, are similar to those described in human disease, where the initial presenting signs are associated with pneumonia or primary lung abscesses (White, 2003).

In addition to the lung, in humans, seeding and abscess formation can arise in any organ; the liver, spleen, skeletal muscle and prostate being common sites (White 2003). A recent fatal case of melioidosis, describes how a male suffered a fever for a week prior to death and at postmortem the lung, liver and spleen contained multiple microabscesses (<1 cm diameter) and neutrophilic infiltration with occasional giant cells (Tan *et al.* 2019), all sites were microbiological culture positive for *B. pseudomallei*. Similary, in the present study, during acute disease, bacteria were found in the spleen, liver, kidney and the highest bacterial burden (3·9 × 10^5^ CFU per mg) was in the lungs of mice, on days 3–4 pc. The pathological changes in the mouse lung, seen here, are similar to those described in human disease (White, 2003). Overall many of the pathogenic characteristics of *B. pseudomallei* NCTC 13392 infection in the present mouse study align with those seen in acute human disease, demonstrating that this bacterial strain, aerosolized dose and mouse model are suitable for evaluating MCMs against acute melioidosis.

The characterisitcs of subacute melioidosis were also observed in the present study when BALB/c mice were exposed to a low dose of NCTC 13392 (<10 CFU per animal). Here, low clinical scores (≤1·2) were observed initially; these subsided until day 20 pc. Afterwards clinical scores began to increase to a mean of 3·2 by day 30 pc, weight loss (4%) was also seen. This pattern of symptoms mimicks human subacute/chronic infection; in humans, chronic melioidosis is characterized as a symptomatic infection that lasts >2 months; the nonspecific signs and symptoms, during the incubation period, can often hinder the diagnosis and treatment of this disease (Wiersinga *et al.* 2018). In this study, on day 10 pc, hepatic abscesses were present and hepatic degeneration and inflammation was observed, which is consistent with subacute/chronic infection. On day 30 pc, bacteria were found in multiple sites and microscopic lesions (pyogranulomas/abscesses), attributable to *B. pseudomallei* infection, were noted in most mice. This is consistent with human subacute/chronic disease, where abscesses in different organs are seen (White, 2003). In the present proof of concept study a model for subacute human infection is described, and thus therapeutics could be prescreened to assess their effectiveness against subacute/chronic disease. The model requires further refinement and would benefit from larger animal numbers per treatment group to enable statistical significance and also a longer study duration (e.g. 60 days).

In the present pathogenesis study, BALB/c survivors exposed to the low and high dose showed clinical signs, histological features of infection and weight loss by day 30; these are clearly indicators of a subacute infection. If this study was longer in duration then these animals may have fully succumbed to the disease and died. The subacute characteristic of *B. pseudomallei* NCTC 13392 has made the accurate estimation of an LD_50_ for this strain difficult to asertain. Using the virulence study data (14 day duration) an estimated LD_50_ of approximately 200 CFU could be determined; however, the data obtained from the 30‐day duration pathogenesis experiment indicates that an LD_50_ of <8 CFU should be used for NCTC 13392. The true LD_50_, however, can only be found by performing a study with a longer observation period (e.g. 60–100 days). Other researchers have also noted a lowering of LD_50_ values, in mice, when they compared the LD_50_ results from 21‐day and 60‐day duration intraperitoneal infection studies with other strains of *B. pseudomallei* (Welkos *et al.* 2015). Recent low‐dose, aerosol challenge studies comparing different clinical isolates of *B. pseudomallei* in BALB/c mice describe an LD_50_ value of 25·1 CFU for K96243 (prototype strain), 0·99 CFU for HBPUB10134a and 0·35 CFU for MSHR5855 (Trevino *et al.* 2018). A working hypothesis is that the virulence of NCTC 13392 falls somewhere between these strains. A property of NCTC 13392 in particular, is that the LD_50_ is likely to be inversely proportional to the study length due to its subacute characteristics, at low dose. The bacterial burden in the lungs and spleens of BALB/c mice infected with HBPUB10134a and MSHR5855 (delivered by aerosol with doses 6 CFU and 1·2 CFU respectively) is higher than mice infected with a low dose (8 CFU) of NCTC 13392 at day 10 pc, indicating that the infection caused by these other two strains, at low doses, becames established more quickly (Trevino *et al.* 2018).

Currently most MCMs for melioidosis are evaluated in animal models for their effectiveness against acute disease; the mouse model being the most widely used (Warawa, 2010) although larger animal models have been evaluated for instance: Rhesus macaques (Yingst, Facemire *et al*. 2014); African Green monkeys (Yeager *et al.* 2012) and marmosets (Nelson *et al.* 2011; Nelson *et al.* 2014; Nelson *et al.* 2015). Aerosolized doses of <10 CFU per animal *B. pseudomallei* NCTC 13392 caused a lethal infection by day 3–4 in marmosets, indicating these animals are highly susceptible to this strain (Nelson *et al.* 2011; Erratum, 2013). Overall there is a need to focus on defining acute disease and developing MCMs because of the potential for *B. pseudomallei* to be used as a biological weapon (Kwon *et al.* 2018). Studies on aerosol delivery are particularly important to help characterize the natural history of inhalational exposure to *B. pseudomallei* in a deliberate‐release scenario, where the inoculum is likely to be much higher than exposure from an environmental source (Gilad *et al.* 2007). Fewer MCMs are tested for their effectiveness against chronic disease and it remains unclear whether treatment recommendations for this condition remain the same as for acute melioidosis (Currie *et al.* 2000; Nandi and Tan, 2013). Chronic disease is usually a public health concern often arising from a low‐dose environmental exposure in a country where this disease is endemic. There are fewer animal models for subacute/chronic melioidosis (Warawa, 2010; Conejero *et al.* 2011; Soffler *et al.* 2012; Amemiya *et al.* 2015; Bearss *et al.* 2017).

In conclusion, the present proof of concept study has, for the first time, shown that *B. pseudomallei* NCTC 13392 is virulent and lethality is dose dependant, when delivered by aerosol to BALB/c mice; thus, this strain is suitable for use in MCM evaluation studies. Both acute and subacute melioidosis disease can also be modelled with this strain by adjusting the challenge dose. Following further refinement, this combination of mouse strain, bacterial strain and dosing regime, will provide a useful toolkit for the preliminary screening of the effectiveness of novel vaccines or therapeutics for both acute and subacute melioidosis, at the same time.

## Conficts of Interest

No conflicts of interest declared.
